# Magnesium Alginate in Gastro-Esophageal Reflux: A Randomized Multicenter Cross-Over Study in Infants

**DOI:** 10.3390/ijerph17010083

**Published:** 2019-12-20

**Authors:** Maria Elisabetta Baldassarre, Antonio Di Mauro, Maria Cristina Pignatelli, Margherita Fanelli, Silvia Salvatore, Giovanni Di Nardo, Andrea Chiaro, Licia Pensabene, Nicola Laforgia

**Affiliations:** 1 Department of Biomedical Science and Human Oncology, Neonatology and Neonatal Intensive Care Unit, “Aldo Moro” University of Bari, 70124 Bari, Italy; antonio.dimauro@uniba.it (A.D.M.); m.pignatelli3@studenti.uniba.it (M.C.P.); nicola.laforgia@uniba.it (N.L.); 2 Department of Interdisciplinary Medicine, “Aldo Moro” University of Bari, 70100 Bari, Italy; margherita.fanelli@uniba.it; 3 Department of Pediatrics, “F. Del Ponte” Hospital, University of Insubria, 21100 Varese, Italy; silvia.salvatore@uninsubria.it; 4 Chiar of Pediatrics, NESMOS Department, School of Medicine and Psychology, Sapienza University of Rome, Sant’Andrea University Hospital, Via di Grottarossa 1035-1039, 00189 Rome, Italy; giovanni.dinardo@icloud.com; 5 Department of Pediatrics, “Maggiore” Hospital, 26013 Crema, Italy; andreachiaro@tiscali.it; 6 Department of Medical and Surgical Sciences, Pediatric Unit, University “Magna Graecia” of Catanzaro, 88100 Catanzaro, Italy; pensabene@unicz.it

**Keywords:** infant regurgitation, magnesium alginate, thickened formula, breastfeeding

## Abstract

The aims of this study were to evaluate the efficacy of magnesium alginate in decreasing functional regurgitation symptoms in infants, and to assess the cost–benefit ratio of magnesium alginate compared to a thickened formula. A multicenter perspective cross-over study was conducted in formula-fed infants with persisting regurgitation, randomly assigned to receive two weeks of a magnesium-alginate-based formulation followed by two weeks of thickened formula, or vice-versa. Infants, exclusively breast-fed, were followed up for two weeks while receiving magnesium alginate. Symptoms of gastroesophageal reflux (GER) were evaluated through the Infant Gastroesophageal Reflux Questionnaire Revised (I-GERQ-R). Direct cost of treatments was also calculated. Seventy-two infants completed the study. We found a significant reduction of I-GERQ-R scores over time (F = 55.387; *p* < 0.001) in all groups with no difference between the sequences of administration (F = 0.268; *p* = 0.848) in formula-fed infants and between exclusively breast-fed and formula-fed infants receiving magnesium alginate (t = 1.55; *p* = 0.126). The mean cost savings per infant was € 4.60 (±11.2) in formula-fed infants treated with magnesium alginate compared to thickened formula (t = 2.91, *p* < 0.0005). Conclusions were that the magnesium-alginate formulation reduces GER symptoms both in formula-fed and breast-fed infants. In formula-fed infants, clinical efficacy is similar to thickened formulas with a slightly lower cost of treatment.

## 1. Background

Infant regurgitation is the most common functional gastrointestinal disorder (FGID) in infants (<12 months of age), with a wide range of prevalence reported from 3% to 87%, depending on the population recruited (geographical origin and age of infants), frequency per day, and length of follow-up [1,2,3,4,5].

Although functional regurgitation resolves spontaneously before the first year of life and therapeutic inactivity should be considered the ultimate goal, it causes frequent parental concern. FGID is responsible for relevant direct and indirect costs from medication, visits, and diagnostics [6,7].

Reassurance and a behavioral approach are suggested as first steps in FGID management [8]. Nutritional advice and formula changes are also considered to be beneficial in infants with persisting symptoms, whereas pharmacological treatment is not recommended [9].

Thickened formulas [10] and alginate-based formulations are often started in infants with persisting regurgitation without typical gastroesophageal reflux disease (GERD) symptoms, such as vomiting, crying, irritability, poor weight gain, and food refusal. Alginate-based formulations from brown algae produce a physical barrier on the top of the stomach contents in the form of a neutral floating gel or raft. Recently, an Italian survey conducted on a large group of adult patients with GERD demonstrated a significant improvement of symptoms [11,12], but limited clinical data are available in infants [13].

The main aims of our study were to evaluate the efficacy of a magnesium-alginate-based formulation in decreasing persistent functional regurgitation symptoms in infants and to assess the cost–benefit ratio of the alginate treatment compared to a thickened formula in formula-fed infants.

## 2. Methods

### Study Design and Patients

This was a multicenter perspective, two-treatment crossover study conducted between January 2016 and December 2018 in infants with regurgitation, referring to four pediatric Italian hospitals.

The Ethical Committee approved the study and written informed consent was obtained from all participating parents. No investigations, such as multichannel intraluminal impedance, pH meter, or endoscopy were scheduled for this population.

The study was registered at ClinicalTrials.gov (NCT02806453). Inclusion criteria were represented by consecutive infants (aged 3 weeks to 4 months) with at least two episodes of regurgitation per day for 3 or more weeks, according to Rome IV criteria (Appendix A) for FGIDs in infancy [14]. Exclusion criteria were one or more of the following: 1) hematemesis; 2) aspiration; 3) apnea; 4) failure to thrive; 5) feeding or swallowing problems; 6) abnormal posturing; 7) abnormal physical or neurological examination; 8) malformation; 9) previous surgery; 10) signs of acute infections; 11) known or suspected metabolic, hepatic, renal or other chronic disease; 12) previous or current use of thickened formulas or complementary feeding; 13) previous diagnosis or suspicion signs of cow’s milk protein allergy (i.e., atopic dermatitis); and 14) previous treatment with alginate or H2-receptor antagonists or proton pump inhibitors (PPIs). Data regarding the neonatal period were also considered and collected at baseline. All parents of enrolled newborns entered in a run-in period of one week. After reassurance on the benign nature of the condition, parents were educated with oral and written instructions to manage this condition by avoiding overfeeding, incorrect positioning, and passive smoking, in accordance with the European Societies for Paediatric Gastroenterology, Hepatology and Nutrition (ESPGHAN) [15] and the National Institute for Health and Care Excellence (NICE) guidelines [16]. Breastfeeding was promoted, supported, and assessed by a person with appropriate expertise. One week after enrolment, parents answered a questionnaire called the Infant Gastro-Esophageal Reflux Questionnaire Revised (I-GERQ-R). The I-GERQ-R is a brief, 12-item, caregiver-completed measure of infant GERD symptoms that has been validated to differentiate cases from infants without sufficient symptoms for the diagnosis, to monitor treatment outcomes in clinical practice, and to serve as an evaluative tool in clinical trials. [17]. We considered persistent regurgitation when I-GERQ-R was above the cut-off limit or normal (≥16), as already reported [18,19].

Infants with I-GERQ-R ≥ 16 were recruited for the intervention study. Formula-fed infants were randomly assigned to receive two weeks of magnesium-alginate supplementation (Refluxsan Nipio^®^, Aurora Biofarma, Milano, Italy) and two weeks of a thickened formula (Group A), or vice versa (Group B). A washout period of one week without any intervention (thickened formula or magnesium-alginate supplementation) was scheduled, as the dynamics of the two treatments were based only on daily consumption. A two-week study period was chosen to ensure high protocol adherence from parents.

Since it was a spontaneous study and milk companies at no point contacted or interfered with any part of this trial, and due to a lack of evidence in a clear superiority of a specific thickener agents, the choice of the commercial infant thickened formula was left to the parents, whereas the appropriate volume of feeding was provided for each infant [10].

The daily amount of thickened formula was calculated to cover normal volume and caloric requirement (150 mL/kg/day). During the two weeks on alginate, all infants continued their previous formula without any other intervention or variation in feeding volume and frequency.

We tested this specific alginate formulation based on its composition (magnesium alginate, xantana rubber, sucralose, sodium methyl para-hydroxybenzoate, and purified water); the dose recommended was 1 mL/kg/day divided over the number of meals, administered 10 min after each feeding, as recently reported in another study assessing alginate efficacy in infants [20].

Randomization was performed using a computer-generated two-treatment allocation sequence (nQuery Advisor v.7.0 software, Statistical Solutions Ltd., Cork, Ireland). To avoid disproportionate numbers of patients in each group, a randomization scheme was performed in blocks of four participants. Based on the study design it was not possible to have a blind assessment or a comparison with placebo. The parents were not aware of the primary outcome of the study or that the I-GERQ-R would have been repeated at follow-up visits.

**Figure 1:**
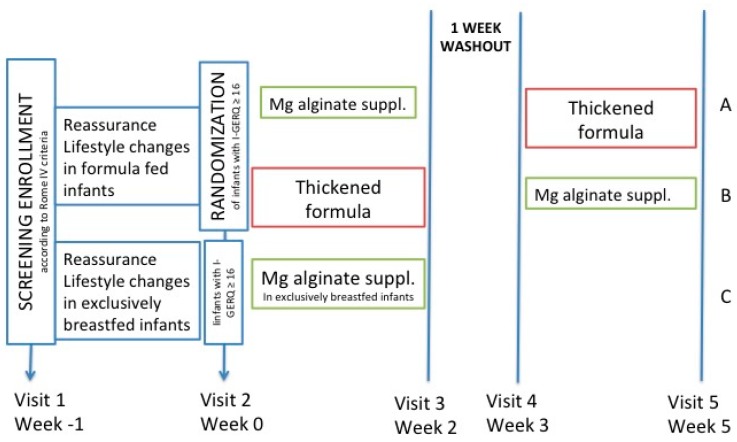
Study design.

Exclusively breast-milk-fed infants with an I-GERQ-R ≥ 16 (Group C) received only two weeks of the magnesium-alginate-based formulation as treatment (Figure 1).

Parents were also instructed to avoid any other treatments, such as probiotics, prokinetics, or acid inhibitors, or to start any variation in coping, position, or infant’s diet during the study.

Anthropometric measures and I-GERQ-R were repeated at the end of each treatment period. The primary outcome of the study was GER symptom score reduction as evaluated by I-GERQ-R and comparison of responder rates among the three groups. Success rate was defined as the proportion of infants reaching an I-GERQ-R < 16 after treatment.

As a secondary aim, we assessed the direct costs of thickened formula and the magnesium-alginate formulation. Direct cost was calculated according to the quantity of thickening formula and alginate-based formulation used during the two-week period, based on the price on the Italian market (on average 5.20 euro/L for thickening formula, 2.36 euro/L for standard formula, and 18.90 euro for 150 mL of Refluxsan Nipio syrup). Indirect cost was not analyzed.

Any significant clinical adverse effects or problems in administering the alginate formulation were also recorded.

## 3. Statistical Analysis

The sample size was calculated using Powerandsamplesize.com© (2013–2019 HyLown Consulting LLC Atlanta, GA, USA) for comparison of two percentages based on data observed in a previous study assessing the efficacy of magnesium alginate in infants with GER [18]. Setting a level of significance 5% and a power of 80%, the sample size requested for each treatment group was 26. Assuming a 20% dropout between screening and randomization, we calculated that a minimum of 70 infants had to be enrolled. Categorical variables were analyzed by a chi-square test.

A one-way analysis of variance test (ANOVA) was performed to compare the three groups. Post-hoc tests were used when necessary. A repeated measure ANOVA with interaction was performed to analyze the crossover design. The cumulative effect of the magnesium-alginate formulation or thickened formula was evaluated by paired Student’s *t*-tests. Cost saving were also analyzed by paired Student’s *t*-tests.

## 4. Results

Ninety-six infants satisfied the inclusion and exclusion criteria. After one week of behavior and nutrition advice, 24 (25%) infants reported a significant improvement of symptoms according to parents and had a normal I-GERQ-R. The other 72 presented persistent symptoms and abnormal I-GERQ-R and were recruited in the intervention phase. Fifty-three were formula-fed and entered the randomization process for the two different treatments: 27 had magnesium-alginate—thickened formula sequence (Group A), and 26 had the thickened formula—magnesium alginate sequence (Group B). The remaining 19 exclusively breast-fed infants were given magnesium-alginate supplementation (Group C).

**Figure 2:**
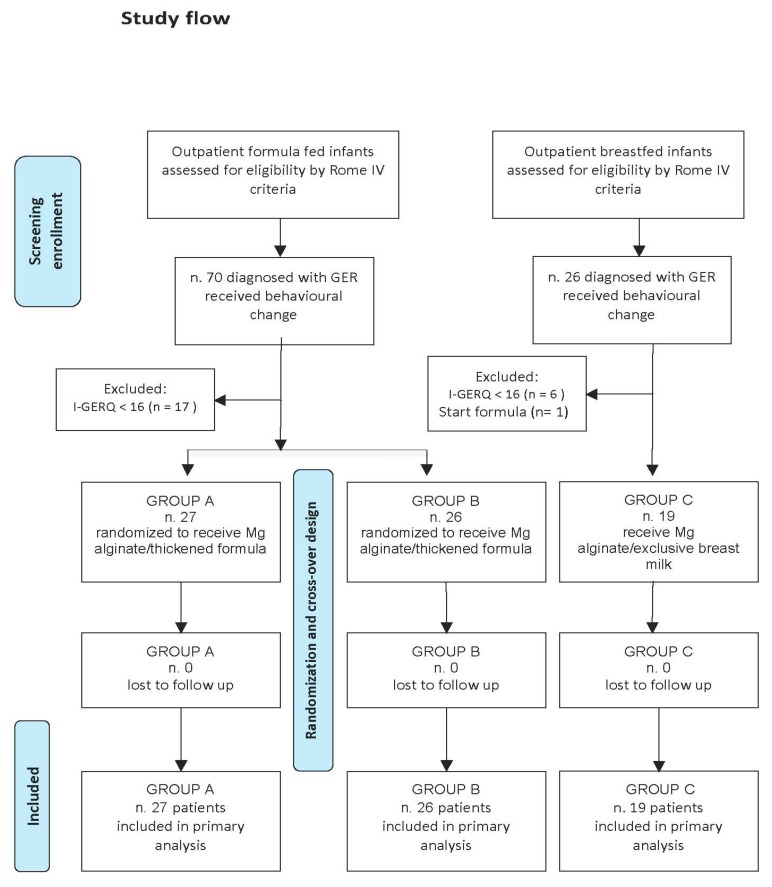
Study flow. GER: gastroesophageal reflux; I-GERQ-R: Infant Gastro-Esophageal Reflux Questionnaire.

Final analysis was performed in 72 infants (Figure 2).

The distribution of infants treated in groups A and B was not statistically different among the four centers (chisquare = 2.296; *p* = 0.513).

Demographic characteristics at enrolment and comparisons among groups are reported in Table 1.

The mean gestational age at birth was not statistically different among groups (F = 1.697; *p* = 0.191).

The mean age at enrolment was 65.5 ± 46.3 days, with a slightly significant difference among groups (F = 3.327; *p* = 0.042). In particular, there was little difference in the mean age at enrolment between groups A and B (Sheffè post hoc test: A vs. B, *p* = 0.049). Mean I-GERQ at enrolment was 22 ± 4 with no significant differences among groups (F = 1.455; *p* = 0.24).

All groups were similar according to sex distribution, mode of delivery, and birth weight (data not shown).

A significant variation of I-GERQ-R scores over time (visits 2–3–4–5) (F = 55.387; *p* < 0.001) were evident, independent of the sequence of administration (magnesium-alginate—thickened formula or thickened formula—magnesium-alginate) (interaction effect: F = 0.268; *p* = 0.848).

Several thickened formulas were used without a significant difference between them (data not shown). Both cumulative effects of magnesium alginate and thickened formula were evaluated, and the mean symptoms score was significantly decreased in both groups (Table 2).

In formula-fed infants no significant differences in I-GERQ-R score reduction between magnesium-alginate cumulative effect and thickened formula cumulative effect were found (*t* = 0.712, *p* = 0,48).

In exclusively breast-fed infants, magnesium alginate was as effective as in formula-fed infants (*t* = 1.55; *p* = 0.126).

After treatment, the I-GERQ-R score normalized in 73% of formula-fed infants treated with alginate, in 64% of formula-fed infants treated with thickening formulas, and finally in 84% of breast-fed infants treated with alginate (chisquare = 2.23, *p* = 0.328).

A significant mean cost saving of 4.60 (±11.2) euro per child was found during the two-week treatment period with alginate (*t* = 2.91, *p* < 0.0005).

## 5. Discussion

Infants with persistent regurgitation not improving after behavioral advice and correction of feeding intake, even while presenting additional symptoms determining a pathological I-GERQ-R, may benefit equally from thickened formula and magnesium-alginate supplementation. A significant decrease of GER symptoms was observed during magnesium-alginate supplementation in both exclusively breast-fed and formula-fed infants.

Presently, sodium alginate is the most studied formulation [20,21,22], with limited concerns regarding sodium uptake [23] bezoar formation [24] in the stomach, and constipation [25] for the patients, but data on its efficacy on GER symptoms in infancy are limited [13].

To the best of our knowledge, only a randomized controlled trial by Ummarino et al. reported significant symptoms reduction by a validated questionnaire, using a magnesium-alginate-based plus simethicone formulation in 64 infants [20]. However, this previous trial was a parallel study comparing three different groups of infants using a highly (14%) thickened formula.

Our findings are consistent with the results of this previous study, although our effect appeared to be related more closely to the alginate compound, as Ummarino used a different alginate formulation enriched with simethicone that, reducing gas in the stomach, might facilitate gastric emptying and possibly related symptoms reduction. Furthermore, the alginate formulation used in Ummarino’s study also contained sodium bicarbonate and fructose, for which the efficacy and safety in children is not yet clarified [26,27]. The magnesium-alginate formulation tested in our trial did not include antacid compounds, but did contain sucralose, considered as a safe, non-caloric, high-intensity sweetener, as an additive [28].

Very recently, Salvatore et al. demonstrated that alginate-based formulations reduced both acid and nonacid reflux episodes assessed by esophageal pH impedance and symptoms reported during the investigation in 31/40 (77%) infants with persisting GER symptoms non-responsive to behavior and diet modification [19].

The ESPGHAN and the North American Societies for Pediatric Gastroenterology, Hepatology and Nutrition (NASPGHAN) guidelines do not recommend use of alginate formulations in GER because of limited studies, whereas NICEs suggest alginate formulations only when behavioral approaches and thickened formulas have failed [15,16].

Based on our clinical data and previous findings, alginate-based formulations may be considered as a first treatment option for persistent and distressing regurgitation and related symptoms in both breast-fed and formula-fed infants.

A pathological I-GERQ-R score in infants does not exclude the possibility of response to alginate formulations or thickened formulas that could be attempted for a period of two weeks before considering additional investigations for GER disease in otherwise healthy infants.

In infants with troublesome symptoms of GER, when a conservative approach has failed, an alginate-based formulation should be preferred to acid inhibitors. These acid inhibitors continue to be over-prescribed despite their cost, the very limited clinical evidence of reducing GER symptoms in this age group, and the possible adverse effects [29].

Magnesium alginate can be effectively and safely used in exclusively or partially breast-fed and formula-fed infants, and may reduce the use of other GER treatments and tests.

Thickened formulas that are available on the market are also a beneficial option for infants with persistent regurgitation, although they may differ in the kind of protein, thickening agent, quantity of lactose, prebiotic, or other components influencing cost and possible clinical efficacy [8]. Thickened formulas are well balanced in terms of both caloric and macro and micronutrient content. They may reduce the use of home-brew thickening agents which may increase caloric intake, possible overweightness, and obesity [30].

We are aware of some limitations of our study. First of all, our results are based on an open study and validated questionnaire with no diagnostic test. Therefore, we cannot exclude the possibility that parents may have under or over-reported symptoms, or a possible placebo effect of both intervention and visits.

Furthermore, it would have been quite interesting to have a control group with only parental guidance, education, and support.

In addition, in breast-fed infants, we did not use a placebo compound, and spontaneous improvement of reflux symptoms cannot be excluded, even if not reported within the previous two weeks. Lastly, the data provided by our study are based on a small population of infants with only short-term follow-up.

Finally, although efficacy trials imply the study of a homogenous population, and our results indicated there was a significant difference of age between the groups at enrolment, the crossover-randomized design should have limited the influence of confounding covariates, since each patient served as their own control.

## 6. Conclusions

Magnesium-alginate formulations reduced persistent GER symptoms both in formula-fed and breast-fed infants not responsive to behavioral and diet approaches. In formula-fed infants, clinical efficacy was similar to thickened formulas with a slightly lower treatment cost. Further well-designed and large double-blind prospective studies are needed to confirm the clinical and cost effectiveness of magnesium-alginate formulations as treatments of troublesome regurgitation in infants.

## Figures and Tables

**Table 1:** Demographic characteristics at enrolment.

	Group A Magnesium-Alginate—Thickened Formula (*n* = 27)	Group B Thickened Formula—Magnesium-Alginate (*n* = 26)	Group C Magnesium Alginate in Exclusively Breast-Fed Infants (*n* = 19)	F	*p*
Gestational age at birth, mean ± SD, weeks	37.3 ± 3.3	36.8 ± 4.0	38.7 ± 1.9	1.882	0.16
Age at enrolment, mean ± SD, days	51.8 ± 41.1	84.4 ± 57.2	60.6 ± 29.6	3.327	0.042
I-GERQ-R at enrolment, mean ± SD	21.2 ± 4.1	22.9 ± 4.8	20.8 ± 4.1	1.455	0.24

**Table 2:** Cumulative effects of each treatment in I-GERQ-R mean reduction in each study group.

	Magnesium Alginate Cumulative Effects in Formula-Fed Infants	Thickened Formula Cumulative Effects in Formula-Fed Infants	Magnesium Alginate in Exclusively Breast-Fed Infants
I-GERQ-R mean reduction	−8.96 (6.93) *t* = 8.77 *p* < 0.0001	−9.74 (7.66) *t* = 8.63 *p* < 0.0001	−10.95 (3.37) *t* = 14.14 *p* < 0.0001

## Abbreviations

ESPGHAN	European Society for Paediatric Gastroenterology, Hepatology and Nutrition
FIGDs	Functional Gastrointestinal Diseases
GER	Gastroesophageal Reflux
I-GERQ-R	Infant Gastroesophageal Reflux Questionnaire Revised
NASPGHAN	North American Society for Pediatric Gastroenterology, Hepatology and Nutrition
NICE	National Institute for Health and Care Excellence
PPI	Proton Pump Inhibitor

## Appendix A. ROME IV Diagnostic Criteria for Infant Regurgitation

Must include both of the following in otherwise healthy infants 3 weeks to 12 months of age:

1. Regurgitation two or more times per day for three or more weeks.
2. No retching, hematemesis, aspiration, apnea, failure to thrive, feeding or swallowing difficulties, or abnormal posturing.
